# Kososan, a Kampo medicine, prevents a social avoidance behavior and attenuates neuroinflammation in socially defeated mice

**DOI:** 10.1186/s12974-017-0876-8

**Published:** 2017-05-03

**Authors:** Naoki Ito, Eiji Hirose, Tatsuya Ishida, Atsushi Hori, Takayuki Nagai, Yoshinori Kobayashi, Hiroaki Kiyohara, Tetsuro Oikawa, Toshihiko Hanawa, Hiroshi Odaguchi

**Affiliations:** 1 Department of Clinical Research, Oriental Medicine Research Center, Kitasato University, Tokyo, Japan; 2 Graduate School of Infection Control Sciences, Kitasato University, Tokyo, Japan; 3 Laboratory of Pharmacognosy, School of Pharmacy, Kitasato University, Tokyo, Japan; 4 Graduate School of Medical Sciences, Kitasato University, Kanagawa, Japan; 5 Laboratory of Biochemical Pharmacology for Phytomedicines, Kitasato Institute for Life Sciences, Kitasato University, Tokyo, Japan

**Keywords:** Kampo, Kososan, Depression, Anxiety, Antidepressant-like effect, Chronic social defeat stress, Microglia, Neuroinflammation, Neurogenesis

## Abstract

**Background:**

Kososan, a Kampo (traditional Japanese herbal) medicine, has been used for the therapy of depressive mood in humans. However, evidence for the antidepressant efficacy of kososan and potential mechanisms are lacking. Recently, it has been recognized that stress triggers neuroinflammation and suppresses adult neurogenesis, leading to depression and anxiety. Here, we examined whether kososan extract affected social behavior in mice exposed to chronic social defeat stress (CSDS), an animal model of prolonged psychosocial stress, and neuroinflammation induced by CSDS.

**Methods:**

In the CSDS paradigm, C57BL/6J mice were exposed to 10 min of social defeat stress from an aggressive CD-1 mouse for 10 consecutive days (days 1–10). Kososan extract (1.0 g/kg) was administered orally once daily for 12 days (days 1–12). On day 11, the social avoidance test was performed to examine depressive- and anxious-like behaviors. To characterize the impacts of kososan on neuroinflammation and adult neurogenesis, immunochemical analyses and ex vivo microglial stimulation assay with lipopolysaccharide (LPS) were performed on days 13–15.

**Results:**

Oral administration of kososan extract alleviated social avoidance, depression- and anxiety-like behaviors, caused by CSDS exposure. CSDS exposure resulted in neuroinflammation, as indicated by the increased accumulation of microglia, the resident immune cells of the brain, and their activation in the hippocampus, which was reversed to normal levels by treatment with kososan extract. Additionally, in ex vivo studies, CSDS exposure potentiated the microglial pro-inflammatory response to a subsequent LPS challenge, an effect that was also blunted by kososan extract treatment. Indeed, the modulatory effect of kososan extract on neuroinflammation appears to be due to a hippocampal increase in an anti-inflammatory phenotype of microglia while sparing an increased pro-inflammatory phenotype of microglia caused by CSDS. Moreover, reduced adult hippocampal neurogenesis in defeated mice was recovered by kososan extract treatment.

**Conclusions:**

Our findings suggest that kososan extract prevents a social avoidant behavior in socially defeated mice that is partially mediated by the downregulation of hippocampal neuroinflammation, presumably by the relative increased anti-inflammatory microglia and regulation of adult hippocampal neurogenesis. Our present study also provides novel evidence for the beneficial effects of kososan on depression/anxiety and the possible underlying mechanisms.

**Electronic supplementary material:**

The online version of this article (doi:10.1186/s12974-017-0876-8) contains supplementary material, which is available to authorized users.

## Background

Long-lasting exposure to various stressors in humans often causes psychological disorders such as depression. Depression per se is not a life-threatening illness, although long-lasting depression could lead to various detrimental events including suicide [1], worsening of other diseases [2], and long-term absence from work [3], which can result in robust economic loss. Therefore, an effective treatment of depression is an urgent social and medical issue.

To date, a large body of research has attempted to elucidate the pathogenesis of depression by using animal models exposed to stressors. Among them, chronic social defeat stress (CSDS) is a psychosocial stress behavioral paradigm widely used with face, constructive, and predictive validity [4–6] and is currently used to study several psychiatric disorders and their pathologies. For example, CSDS is used to examine depression, anxiety, and the efficacy of therapeutic drugs to treat these conditions [7–9].

Recently, it has been well recognized that stress induces neuroinflammation via microglia, the resident immune cells of the brain [10–14], which potentially contributes to the onset of depression [15] and anxiety [16]. Interestingly, the classical tricyclic antidepressant imipramine prevents neuroinflammation and behavioral deficits (depression- and anxiety-like behaviors) caused by CSDS [17, 18]. In addition, studies without stress exposure found that interferon-alpha (IFN-α) therapy elicited neuroinflammation as well as depression-like behaviors [19], both of which were prevented by minocycline, an inhibitor of microglial activation [20]. Microglial activation is also well known to be categorized into the classical M1 and alternative M2 phenotypes [21–23]. M1 microglia produce pro-inflammatory cytokines (i.e., IL-1β, IL-6, TNF-α), inducible nitric oxide, and reactive oxygen species that lead to cell damage. M2 microglia serve as an anti-inflammatory phenotype that is involved in tissue repair and remodeling. Thus, M1 and M2 microglia are considered to act as inducers and suppressors of neuroinflammation, respectively. A more recent study has shown that pioglitazone, a highly selective agonist for peroxisome proliferator-activated receptor γ (PPARγ), exerts an antidepressant-like activity through PPARγ-mediated amelioration of M1/M2 microglial imbalance in chronic mild stress-induced depression-like model mice [24]. Taken together, these findings suggest that microglia-mediated neuroinflammation could be a therapeutic target in treating depression and anxiety.

Kososan, a Kampo (traditional Japanese herbal) medicine, is composed of five herbs (Cyperi Rhizoma, Perillae Herba, Aurantii Nobilis Pericarpium, Glycyrrhizae Radix, and Zingiberis Rhizoma). Experientially, kososan is currently used to treat depressive mood disorders in addition to the initial stage of the common cold, allergic urticaria due to the ingestion of food, irritable bowel syndrome, chronic fatigue syndrome, insomnia, and autonomic imbalance. There is also modern clinical evidence showing that kososan attenuates depressive mood caused by IFN-α therapy for hepatitis C [25]. Moreover, our previous animal studies have demonstrated that oral administration of kososan counteracted the depression-like behaviors of chronic mild stress-exposed or IFN-α-treated mice by normalizing the dysfunction of the hypothalamic-pituitary-adrenal axis, a region strongly associated with the pathogenesis of depression [26, 27], regulating the orexin/neuropeptide Y signaling system [28, 29], and by modulating metabotropic glutamate receptor 2 and 2′,3′-cyclic nucleotide 3′-phosphodiesterase 1 in the hypothalamus using a proteomic analysis [30]. Furthermore, our recent study suggests that psychological stress-induced depression-like behaviors in mice were mitigated by treatment with kososan, but not the antidepressant milnacipran [31], a serotonin-noradrenaline reuptake inhibitor. Besides, many rodent studies have shown that some compounds (e.g., apigenin [32, 33], caffeic acid [34], perillaldehyde [35, 36], and rosmarinic acid [37] contained in Perillae Herba; hesperidin [38, 39] and nobiletin [40, 41] contained in Aurantii Nobilis Pericarpium) exert antidepressant-like effects. Although there is increasing evidence for kososan’s therapeutic benefits for depression-like behaviors in preclinical studies, little is known about the efficacy of kososan in the behavioral abnormalities caused by CSDS as an animal model of psychosocial stress. Therefore, in the present study, we examined whether kososan alters a social avoidant behavior and neuroinflammation in mice exposed to CSDS.

## Methods

### Animals

Male C57BL/6J (7 weeks of age) and CD-1 (retired breeders) mice were purchased from Japan SLC (Hamamatsu, Japan). All animals were allowed to acclimate for at least 1 week after arrival. The C57BL/6J mice were housed in cohorts of four to five, and the CD-1 mice were singly housed during acclimation. The animals were kept under a constant temperature (23 ± 2 °C), humidity (55 ± 10%), and a 12-h light cycle (lights on at 08:00), with food (CE-2, CLEA Japan, Inc., Tokyo, Japan) and water available ad libitum. All cages (22.5 × 33.8 × 14 cm, CLEA Japan, Inc.) were provided with wood bedding material (Japan Laboratory Animals, Inc., Tokyo, Japan). All animal experiments were approved by the Institutional Animal Care and Use Committee of Kitasato University and performed in accordance with the Guidelines for the Care and Use of Laboratory Animals of Kitasato University and the National Research Council Guide for the Care and Use of Laboratory Animals in Japan. Every effort was made to minimize the number of animals used and their suffering.

### Preparation of kososan extract

The herbs in kososan were as follows: Cyperi Rhizoma (the rhizome of *Cyperus rotundus* L.), 4.0 g (Lot No. AE7951, Tsumura & Co., Tokyo, Japan); Perillae Herba (leaf of *Perilla frutescens* Britton var. *acuta* Kudo), 2.0 g (Lot No. B04401, Tsumura & Co.); Aurantii Nobilis Pericarpium (pericarp of *Citrus unshiu* Markovich), 3.0 g (Lot No. AD7971, Tsumura & Co.); Glycyrrhizae Radix (root of *Glycyrrhiza uralensis* Fisher), 2.0 g (Lot No. 8661621, Uchida Wakan-yaku Co. Ltd., Tokyo, Japan); and Zingiberis Rhizoma (rhizome of *Zingiber officinale* Roscoe), 0.5 g (Lot No. AK8761, Tsumura & Co.). Kososan was decocted with 600 ml of distilled water until the volume was reduced by half. The water extract was immediately filtered, centrifuged at 1000 × *g* for 10 min at 4 °C, and the supernatant lyophilized. Total yield of kososan extract was approximately 28% from the herbal mixture based on dry weight [26, 29, 31].

### Bromodeoxyuridine (BrdU) injection

**Fig. 1:**
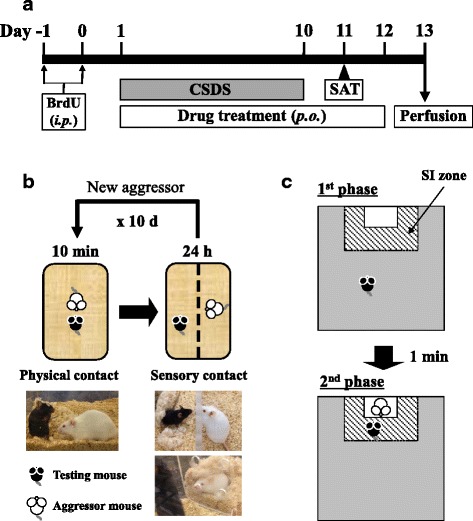
Schematic representation of the experimental schedule (**a**), CSDS procedure (**b**), and SAT procedure (**c**). *BrdU* bromodeoxyuridine, *CSDS* chronic social defeat stress, *SAT* social avoidance test

BrdU (Roche Diagnostics, Indianapolis, IN, USA), a thymidine analog that labels dividing cells in the S-phase of the cell cycle [42], was dissolved in saline with 0.007 N NaOH. BrdU (150 mg/kg, i.p.) was administered once daily for the 2 days prior to the onset of CSDS (Fig. 1a).

### Drug treatment and measurement of body weight

Kososan extract was dissolved in distilled water. Kososan extract (1.0 g/kg) or distilled water was administered by oral gavage once daily for 12 consecutive days (Fig. 1a). The dose of kososan extract (1.0 g/kg) used in this study was chosen based on the findings that kososan extract exhibited an antidepressant-like effect in stress-induced mouse models of depression [26, 28–31]. Body weight was measured prior to kososan extract administration each day.

### CSDS paradigm

CSDS was performed using similar methods described by Krishnan et al. [43] and Golden et al. [4]. Briefly, each testing mouse (C57BL/6J) to be socially defeated was introduced into the home cage of an unfamiliar resident CD-1 aggressor mouse for 10 min daily for 10 consecutive days (days 1–10, Fig. 1a, b). The CD-1 mice were selected and designated as aggressors only if their attack latencies were shorter than 60 s on two to three consecutive screening tests. During the 10-min defeat period, most testing mice showed submissive postures (standing upright) against the aggressor mice. After 10 min of physical contact, the testing mouse and the resident aggressor mouse were each housed in one half of a cage separated by a clear perforated Plexiglas divider to allow sensory contact for the remainder of the 24-h period with free access to food and water. On each testing day, testing mice were defeated by novel aggressor mice to avoid habituation to individual aggressors. Undefeated control mice were handled every day, housed in pairs, separated by the perforated divider in cages identical to those used for socially defeated mice, rotated daily in a manner similar to the defeated mice, but were never exposed to aggressor mice. If mice did not show either submissive postures or flight behaviors during the last exposure of defeat stress, they were excluded as lack of defeat from subsequent experiments. CSDS was carried out between 13:00 h and 17:00 h. The morning after the last defeat session, both defeated and undefeated control mice were individually housed until the end of experiments.

### Social avoidance test (SAT)

The SAT is composed of two 150-s phases [4, 43]. On day 11 (Fig. 1a, c), each mouse was introduced into an opaque gray open-field box (40 × 40 × 40 cm) with an empty wire-mesh Plexiglas enclosure (7 × 10 × 40 cm) located in the social interaction (SI) zone (13.5 × 24.0 cm) at one end of the box and allowed to explore freely for 150 s (the first phase). The mouse was then removed from the box and placed back into its home cage for roughly 1 min. In the second phase, the mouse was re-introduced into the box with an unfamiliar aggressor mouse and allowed to explore again for 150 s. Time spent in the SI zone as well as total distance moved during each phase was recorded by a video tracking system (EthoVision 3.0; Noldus, Wageningen, Netherlands). The SI ratio was calculated by dividing the time spent in the SI zone when the aggressor mouse was present by the time spent in the SI zone when the aggressor mouse was absent. The SAT was carried out between 12:00 h and 17:00 h.

### Brain fixation

On day 13 (Fig. 1a), under deep inhaled anesthesia with isoflurane (Pfizer, Tokyo, Japan), mice were transcardially perfused with cold phosphate-buffered saline (PBS), followed by a cold 4% paraformaldehyde solution (Wako Pure Chemical Industries, Osaka, Japan). The brains were collected and postfixed in a 4% paraformaldehyde solution at 4 °C overnight and then stored in 0.02% NaN_3_/PBS at 4 °C until brain sectioning.

### Immunohistochemistry for ionized calcium binding adaptor molecule 1 (Iba1), CX3C chemokine receptor 1 (CX3CR1), and nod-like receptor family, pyrin domain-containing 3 (NLRP3)

Serial coronal sections (50 μm thick) were obtained throughout the hippocampus using a vibratome (Technical Products International, St. Louis, MO, USA). Staining was completed in 24-well plates for free-floating immunohistochemistry. After incubation with 3% H_2_O_2_/80% methanol for 40 min at room temperature (RT), free-floating sections were blocked for 1 h at RT with blocking buffer [1% bovine serum albumin (BSA; Wako Pure Chemical Industries) in PBS containing 0.3% Triton X-100 (PBS-T)]. Sections were subsequently incubated overnight at 4 °C with rabbit anti-Iba1 (1:1500, Wako Pure Chemical Industries), goat anti-CX3CR1 (1:50, Santa Cruz Biotechnology, Santa Cruz, CA, USA), or mouse anti-NLRP3 (1:1000, AdipoGen, San Diego, CA, USA) primary antibody in the blocking buffer. Sections were then rinsed in PBS-T, incubated for 1 h at RT with biotinylated goat anti-rabbit (1:500; Vector Laboratories, Burlingame, CA, USA), biotinylated donkey anti-goat (1:200, Santa Cruz Biotechnology), or biotinylated horse anti-mouse (1:200, Vector Laboratories) secondary antibody, followed by incubation for 1 h at RT with the ABC kit (Vector Laboratories). Iba1-, CX3CR1-, and NLRP3-positive cells were visualized with Vector DAB (Vector Laboratories). Sections were mounted on silane-coated slides, dried, counterstained with 0.05% toluidine blue (Sigma, St. Louis, MO, USA), dehydrated, and coverslipped. For quantitative assessment, counting of Iba1-positive cells was performed on every third section throughout the hippocampus of both brain hemispheres (bregma −1.5 to −2.6 mm) at ×400 magnification using a light microscope (Olympus BX-41, Olympus Corporation, Tokyo, Japan) in six sections per mouse. Likewise, CX3CR1- or NLRP3-positive cells were also counted on every seventh section throughout the hippocampus of a brain hemisphere, resulting in a total of three sections assessed per mouse.

### Immunofluorescence staining of Iba1/CX3CR1 or NLRP3

Free-floating sections were incubated in 80% methanol for 20 min at RT. Sections were blocked for 1 h at RT and then incubated overnight at 4 °C with rabbit anti-Iba1 (1:1000) and goat anti-CX3CR1 (1:50) or mouse anti-NLRP3 (1:300) primary antibody in the blocking buffer. Sections were then incubated for 1 h at RT with appropriate Alexa Fluor 488- or 594-labeled secondary antibodies (1:1000, Molecular Probes, Eugene, OR, USA). After processing for 30 s with TrueBlack solution (Biotium, Hayward, CA, USA) to quench lipofuscin autofluorescence, sections were coverslipped with VECTASHIELD (Vector Laboratories). Images of double-stained cells in the dentate gyrus were taken at ×400 magnification with a fluorescence microscope (Olympus BX-41) using cellSens imaging software (Olympus Corporation).

### Double immunohistochemistry for BrdU and doublecortin (DCX)

Double immunohistochemistry of BrdU and DCX, a marker of immature neurons, was performed using a two-step staining process as described previously [44, 45].

### Microglia isolation

Microglia were isolated from adult testing mouse whole brain except the cerebellum as described previously [11, 46, 47] with some modifications. Briefly, following decapitation, the whole brain except the cerebellum was readily extracted and chopped finely with a fine sharp scissor in ice-cold serum-free Dulbecco’s modified Eagle’s medium (DMEM)/F12 (Sigma) containing papain (20 U/ml, Worthington Biochemical Corporation, Lakewood, NJ, USA), DNase I (2 mg/ml, Sigma), and 1% penicillin/streptomycin (Invitrogen, Carlsbad, CA, USA). The brain pieces prepared were incubated in a water bath at 37 °C for 20 min. Enzymatic digestion with papain was terminated by adding ice-cold DMEM/F12 containing 20% horse serum (Invitrogen) and 1% penicillin/streptomycin. The brain pieces were further triturated by gently pipetting and passing the tissue through a 100-μm cell strainer (Greiner Bio-One, Tokyo, Japan) to remove cell debris and undigested tissue pieces. The filtered cell suspension was centrifuged at 1000 × *g* for 5 min at 4 °C, and the supernatant was decanted. The cell pellet was then re-suspended by slow pipetting with 30% isotonic Percoll (GE Healthcare, Tokyo, Japan) in Hank’s balanced salt solution without calcium and magnesium (Sigma) and centrifuged at 700 × *g* for 10 min at 4 °C. After centrifugation, the supernatant was aspirated, and the cell pellet was re-suspended by pipetting with a lysis buffer (150 mM NH_4_Cl, 0.24 mM NaHCO_3_, 0.068 mM EDTA in distilled water, pH 7.4) to remove red blood cells and then centrifuged at 1000 × *g* for 5 min at 4 °C. This process was repeated twice to eliminate the remaining dead cells, red blood cells, and Percoll. The cell pellet was re-suspended in DMEM/F12 containing 10% fetal bovine serum (Sigma) and 1% penicillin/streptomycin and filtered through 11-μm nylon mesh (Merck Millipore, Billerica, MA, USA). The harvested cells were counted using a hemocytometer and 0.1% trypan blue solution (Nacalai Tesque, Kyoto, Japan).

### Ex vivo microglial stimulation assay with lipopolysaccharide (LPS)

Microglia were plated at a density of 5 × 10^4^ cells/well in 96-well plates, kept in a 5% CO_2_ incubator at 37 °C for 30 min, and then stimulated with PBS or *Escherichia coli* LPS (serotype 0111:B4, 0.1 μg/ml, Sigma) for 18 h at 37 °C and 5% CO_2_. Supernatants were collected and stored at −80 °C until the interleukin-6 (IL-6) assay. The remaining cells were incubated with 10% alamarBlue (Thermo Fisher Scientific, Waltham, MA, USA) in DMEM/F12 for 2 h at 37 °C and 5% CO_2_, and cell viability was measured using fluoroscopy with the Infinite M200 microplate reader (Tecan Group Ltd., Männedorf, Switzerland) (excitation and emission wavelength at 544 and 590 nm, respectively).

### IL-6 assay

IL-6 levels in supernatants collected from cell cultures were detected using a commercially available solid-phase sandwich ELISA (BD OptEIA^TM^ mouse IL-6 ELISA set, BD Biosciences, San Diego, CA, USA), in accordance with the manufacturer’s instructions. The sensitivity of the measurement was 3.8 pg/ml. The intra- and inter-assay coefficients of variations were 6.4–6.9 and 4–9.6%, respectively.

### Statistics

All data are presented as mean ± standard error of the mean (SEM) and analyzed using Prism (GraphPad Software, San Diego, CA, USA). For comparison between two groups, statistical analysis was performed by unpaired or paired *t* test. For comparison between three or more groups, statistical analysis was performed using a one-way analysis of variance (ANOVA) or two-way repeated measures ANOVA, followed by Bonferroni’s post hoc test. A chi-square test was used for comparison of the binary data from the SAT. In all cases, differences were considered statistically significant at *p* < 0.05.

## Results

### Kososan extract reversed stress-induced social avoidance behaviors in mice

**Fig. 2:**
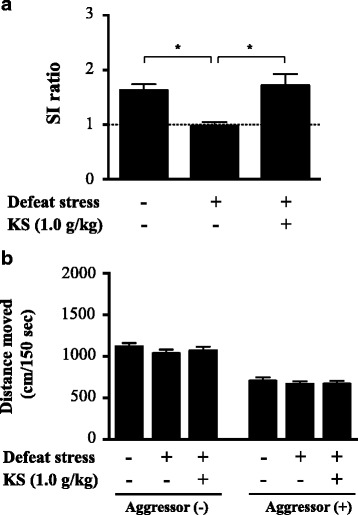
Kososan extract blocked CSDS-induced social avoidance behaviors in mice. Social avoidance test (SAT) was performed on day 11. **a** The social interaction (SI) ratio in the SAT. **b** Total distance moved in the presence or absence of an aggressor mouse during the SAT. Data are presented as the mean ± SEM (*n* = 18–19 per group). *p < 0.01 according to Bonferroni’s post hoc test. *KS* kososan

The SI ratio was significantly lower in socially defeated mice than in undefeated control mice on the SAT (Fig. 2a; one-way ANOVA, *F*_(2,53)_ = 8.064, *p* < 0.001; post hoc test, *p* < 0.01). Kososan extract treatment blocked the stress-induced reduction in the SI ratio (one-way ANOVA, *F*_(2,53)_ = 8.064, *p* < 0.001; post hoc test, *p* < 0.01). Both water-treated undefeated and kososan-treated defeated mice had longer time spent in the SI zone with than without an aggressor (Additional file 1: Figure S1A; *t*_17_ = 6.643, *p* < 0.001; *t*_18_ = 3.23, *p* < 0.01, respectively). But, there was no difference in time spent in the SI zone between with and without an aggressor in the water-treated defeated mice (*t*_18_ = 0.427, *p* = 0.67). Visual tracking data on the SAT confirmed that the mean time spent in the SI zone among water-treated defeated mice was less than both water-treated undefeated and kososan-treated defeated mice when an aggressor was present (Additional file 1: Figure S1B). Mice that showed obvious social avoidant behaviors (i.e., mice with a SI ratio of less than 1) were identified in each group as follows: the water-treated undefeated group (0.56%; 1 out of 18 mice), the water-treated defeated group (57.9%; 11 out of 19 mice), and the kososan extract-treated defeated group (21.1%; 4 out of 19 mice) (water- vs. kososan-treated defeated group, chi-square = 5.397, *df* = 1, *p* < 0.05). These results suggest that kososan extract blocks social defeat stress-triggered social avoidance in mice. In addition, no difference in the total distance moved between groups was seen in each absence (one-way ANOVA, *F*_(2,53)_ = 0.839, *p* = 0.44) and presence (one-way ANOVA, *F*_(2,53)_ = 0.305, *p* = 0.74) of aggressor mouse on the SAT (Fig. 2b). This indicates that the social avoidance behaviors exhibited by each group on the SAT were not simply due to an alteration in locomotor activity. In addition, in kososan extract-treated undefeated mice, neither social avoidance behavior (Additional file 2: Figure S2A) nor total distance moved (Additional file 2: Figure S2B) was affected in the SAT.

**Fig. 3:**
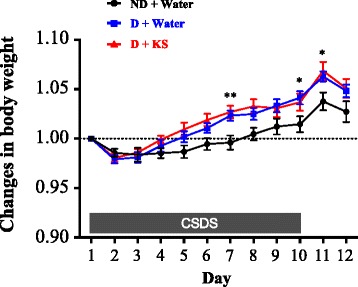
CSDS-induced increase in body weight was not affected by kososan extract treatment. Body weight of mice was recorded daily for 12 days (days 1 through 12) before drug treatment. Changes in body weight each day were calculated based on the deviation from body weight on day 1 (body weight each day/body weight at day 1). Data are presented as the mean ± SEM (*n* = 18–19 per group). *p < 0.05 and **p < 0.01 (D + water vs. ND + water) according to Bonferroni’s post hoc test. *CSDS* chronic social defeat stress, *D* defeated, *ND* non-defeated, *KS* kososan

Body weight of mice was recorded throughout the experiments. Weight in all experimental groups transiently diminished and then gradually increased (Fig. 3; two-way repeated measures ANOVA; time, *F*_(11,583)_ = 81.05, *p* < 0.001; group, *F*_(2,53)_ = 3.802, *p* < 0.05; interaction, *F*_(22,583)_ = 2.56, *p* < 0.001; Additional file 3: Table S1, one-way ANOVA for simple main effects). Particularly after day 4, weight gain among water- and kososan extract-treated defeated mice was greater than that of water-treated undefeated mice over time. Post hoc analysis on individual days revealed that defeat stress induced a significant increase in weight gain relative to undefeated control on days 7 (one-way ANOVA, *F*_(2,53)_ = 7.177, *p* < 0.01; post hoc test, *p* < 0.01), 10 (one-way ANOVA, *F*_(2,53)_ = 3.291, *p* < 0.05; post hoc test, *p* < 0.05), and 11 (one-way ANOVA, *F*_(2,53)_ = 4.316, *p* < 0.05; post hoc test, *p* < 0.05). Temporal change in body weight was not different between water- and kososan extract-treated groups under non-stressed condition (data not shown).

### Kososan prevented stress-induced increases in hippocampal Iba1-positive cells and their aggregates in mice

**Fig. 4:**
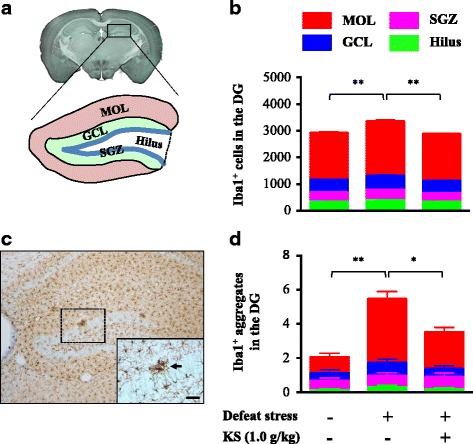
Kososan extract inhibited the stress-induced increase in hippocampal microglia in mice. On day 13, Iba1 staining was performed on six coronal brain sections per mouse. **a** A schematic diagram of the four subregions of the DG in the hippocampus. **b** The count of Iba1-positive cells in the four subregions of the DG. **c** A representative photomicrograph of the DG depicts microglial marker Iba1-positive (*brown-stained*) cells. The *arrow* in the *inset* shows an Iba1-positive aggregate. *Scale bar* = 20 μm. **d** The count of Iba1-positive aggregates in the four subregions of the DG is shown. Data are presented as the mean ± SEM (*n* = 18–19 per group). *p < 0.01 and **p < 0.001 according to Bonferroni’s post hoc test. *MOL* molecular layer, *GCL* granular cell layer, *SGZ* subgranular zone, *DG* dentate gyrus, *KS* kososan

In the dentate gyrus (DG) of the hippocampus (Fig. 4a), the total number of Iba1-positive cells of water-treated defeated mice was significantly higher than that of water-treated undefeated mice (Fig. 4b; one-way ANOVA, *F*_(2,53)_ = 26.94, *p* < 0.001; post hoc test, *p* < 0.001), which was significantly decreased by kososan extract treatment (one-way ANOVA, *F*_(2,53)_ = 26.94, *p* < 0.001; post hoc test, *p* < 0.001). Further, such changes in Iba1-positive cells were similarly found in all regions of the DG [i.e., molecular layer (MOL), granular cell layer (GCL), subgranular zone (SGZ), and hilus] (Additional file 4: Figure S3A; one-way ANOVA; MOL, *F*_(2,53)_ = 21.56, *p* < 0.001; GCL, *F*_(2,53)_ = 6.274, *p* < 0.01; SGZ, *F*_(2,53)_ = 13.54, *p* < 0.001; hilus, *F*_(2,53)_ = 10.78, *p* < 0.001). Likewise, the total number of Iba1-positive aggregates (arrow in Fig. 4c) among water-treated defeated mice was significantly higher than that of water-treated undefeated mice (Fig. 4d; one-way ANOVA, *F*_(2,53)_ = 16.75, *p* < 0.001; post hoc test, *p* < 0.001), which were significantly decreased by kososan extract treatment (one-way ANOVA, *F*_(2,53)_ = 16.75, *p* < 0.001; post hoc test, *p* < 0.01). However, this result was dependent on altered aggregates in the MOL but not the other regions of the DG (Additional file 4: Figure S3B; one-way ANOVA; MOL, *F*_(2,53)_ = 18.32, *p* < 0.001; GCL, *F*_(2,53)_ = 1.003, *p* = 0.37; SGZ, *F*_(2,53)_ = 0.222, *p* = 0.8; hilus, *F*_(2,53)_ = 0.6, *p* = 0.55). In addition, the total number of Iba1-positive cells was positively correlated with that of the Iba1-positive aggregates in the DG (Pearson’s correlation coefficient, *r* = 0.532, *p* < 0.001; data not shown). Under non-stressed condition, kososan extract had no effect on the total number of Iba1-positive cells and their aggregates in the DG (Additional file 2: Figure S2C).

### Kososan extract mitigated the stress-enhanced IL-6 production from isolated microglia

**Fig. 5:**
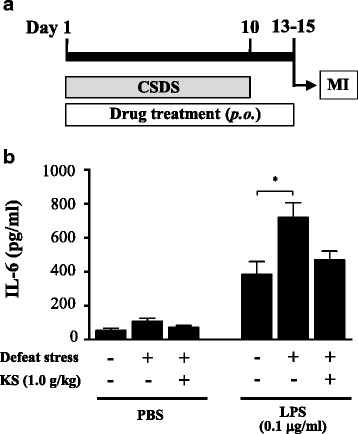
Kososan extract mitigated the stress-enhanced production of IL-6 from isolated microglia. On days 13–15, isolated microglia (5 × 10^4^ cells/well) were stimulated with PBS or lipopolysaccharide (LPS, 0.1 μg/ml) for 18 h and IL-6 levels in the cultured supernatants were examined. **a** The schematic representation of the experimental schedule for MI. **b** IL-6 levels of the supernatants in the absence or presence of LPS are shown. Data are presented as the mean ± SEM (*n* = 6–7 per group). *p < 0.05 according to Bonferroni’s post hoc test. *CSDS* chronic social defeat stress, *MI* microglia isolation, *KS* kososan

Microglia were isolated on days 13–15 from adult mice subjected to exposure to 10 days of CSDS and 13–15 days of treatment with kososan extract (Fig. 5a). Cell viability was slightly reduced by LPS stimulation, but no significant differences were found among the groups (water-treated undefeated mice, 84.8 ± 4.9%; water-treated defeated mice, 83.7 ± 4.4%; kososan extract-treated defeated mice, 89.3 ± 4.9% of PBS-treated controls). Under basal culture conditions (without LPS stimulation), IL-6 levels in the culture media of microglia derived from water-treated defeated mice tended to be higher than those of water-treated undefeated and kososan extract-treated defeated mice, although there were no significant differences in IL-6 levels at baseline (Fig. 5b, left; one-way ANOVA, *F*_(2,17)_ = 3.047, *p* = 0.074). On the other hand, IL-6 released from the microglia of water-treated defeated mice was significantly increased by the LPS challenge relative to that of water-treated undefeated mice (Fig. 5b, right; one-way ANOVA, *F*_(2,17)_ = 5.508, *p* < 0.05; post hoc test, *p* < 0.05). However, IL-6 released from the microglia of kososan extract-treated defeated mice was maintained even after an LPS challenge at a level similar to that of water-treated undefeated mice [Fig. 5b, right; one-way ANOVA, *F*_(2,17)_ = 5.508, *p* < 0.05; post hoc test, *p* = 0.1 (defeat stress + water vs. defeat stress + kososan extract)]. IL-6 levels in the culture media of microglia derived from kososan extract-treated undefeated mice were comparable to those from water-treated undefeated mice (Additional file 2: Figure S2D).

### Kososan extract regulated changes in an anti-inflammatory, but not pro-inflammatory, phenotype of microglia in socially defeated mice

**Fig. 6:**
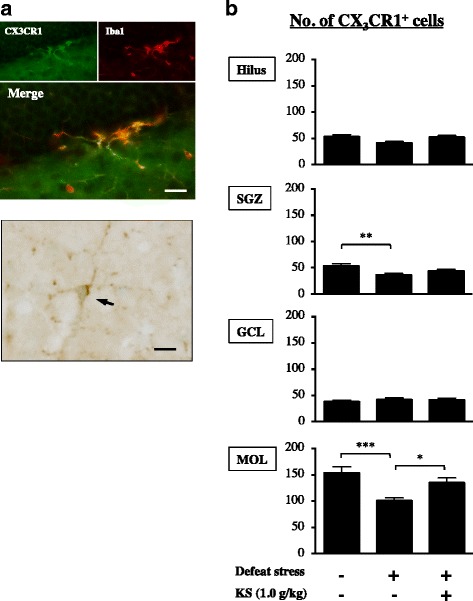
Kososan extract improved the stress-induced decrease in hippocampal CX3CR1-positive cells in mice. On day 13, CX3CR1 staining was performed using three coronal sections per mouse. **a** Representative photomicrographs depicting CX3CR1 co-expressed with an Iba1-positive cell (*upper panel*) and a CX3CR1-positive cell (*lower panel*) in the DG. The *arrow* in the *lower panel* shows a CX3CR1-positive cell in the MOL. *Scale bar* = 20 μm. **b** The number of CX3CR1-positive cells in each of the four subregions of the DG is presented. Data are presented as the mean ± SEM (*n* = 12–15 per group). *p < 0.05, **p < 0.01, and ***p < 0.001 according to Bonferroni’s post hoc test. *MOL* molecular layer, *GCL* granular cell layer, *SGZ* subgranular zone, *DG* dentate gyrus, *KS* kososan

CX3CR1 is predominantly expressed on alternative activated microglia (an anti-inflammatory microglia phenotype) [48–50]. In this study, CX3CR1 expression was found in Iba1-positive microglia in the DG (Fig. 6a). Notably, most of the CX3CR1-positive cells existed in the MOL (51.5%) relative to other DG regions (hilus, 17.8%; SGZ, 17.8%; GCL, 12.9%) in the water-treated undefeated mice (Fig. 6b). The number of CX3CR1-positive cells in the SGZ and MOL of water-treated defeated mice was significantly lower than that of water-treated undefeated mice (Fig. 6b; one-way ANOVA; SGZ, *F*_(2,38)_ = 5.335, *p* < 0.01; post hoc test, *p* < 0.01; MOL, *F*_(2,38)_ = 8.684, *p* < 0.001; post hoc test, *p* < 0.001), which was significantly increased by kososan extract treatment in the MOL but not the SGZ (one-way ANOVA, *F*_(2,38)_ = 8.684, *p* < 0.001; post hoc test, *p* < 0.05).

**Fig. 7:**
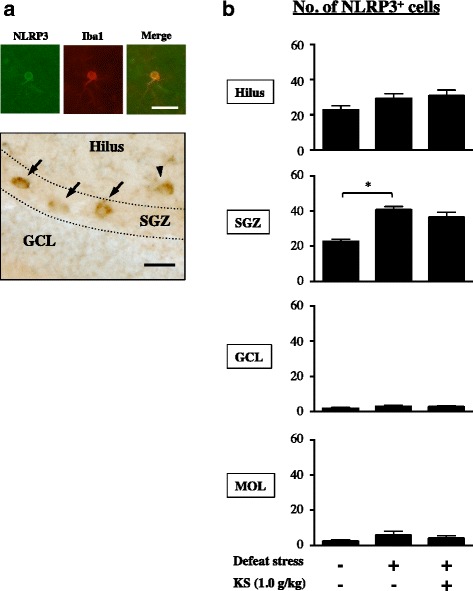
Kososan extract did not improve the stress-induced increase in hippocampal NLRP3-positive cells in mice. On day 13, NLRP3 staining was performed on three coronal brain hemisphere sections per mouse. **a** Representative photomicrographs depict NLRP3 co-expressed with an Iba1-positive cell (*upper panel*) and NLRP3-positive cells (*lower panel*) in the DG. *Arrows* and *arrowhead* in the *lower panel* show NLRP3-positive cells in the SGZ and hilus, respectively. *Scale bar* = 20 μm. **b** The number of NLRP3-positive cells in each of the four DG subregions is presented. Data are shown as the mean ± SEM (*n* = 12–15 per group). *p < 0.001 according to Bonferroni’s post hoc test. *MOL* molecular layer, *GCL* granular cell layer, *SGZ* subgranular zone, *DG* dentate gyrus, *KS* kososan

NLRP3 is predominantly expressed in classically activated microglia (a pro-inflammatory microglia phenotype) [51]. In this study, NLRP3 expression was found in Iba1-positive microglia in the DG (Fig. 7a). Contrary to CX3CR1-positive cells, most of the NLRP3-positive cells were found in the hilus (45.8%) and SGZ (45.6%) relative to the other DG regions (GCL, 3.7%; MOL, 4.9%) in the water-treated undefeated mice (Fig. 7b). The number of NLRP3-positive cells in the SGZ of water-treated defeated mice was significantly higher than that of water-treated undefeated mice (Fig. 7b; one-way ANOVA, *F*_(2,38)_ = 17.52, *p* < 0.001; post hoc test, *p* < 0.001), which was not diminished in kososan extract-treated defeated mice.

### Kososan extract alleviated the stress-elicited reduction in hippocampal neurogenesis

**Fig. 8:**
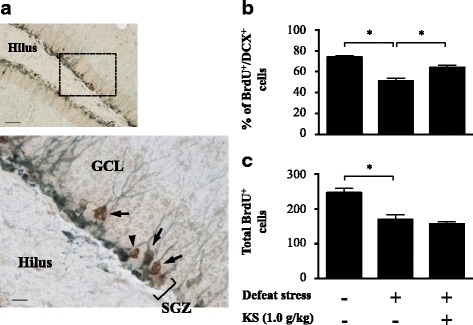
Kososan extract restored the stress-elicited reduction in hippocampal neurogenesis. On day 13, BrdU and DCX double immunostaining was performed on three coronal brain hemisphere sections per mouse. **a** Representative photomicrographs depicting BrdU-positive (*brown-stained*) and/or DCX-positive (*gray-stained*) cells in the SGZ. The *lower panel* shows a high magnification of the selected area in the *upper panel. Arrows* and *arrowhead* in the *lower panel* show the BrdU/DCX double-positive and BrdU-positive/DCX-negative cells, respectively, in the SGZ. *Scale bar* = 50 μm (*upper*) and 10 μm (*lower*). **b** The percentage of BrdU/DCX double-stained cells in the SGZ is calculated by dividing the number of BrdU/DCX double-stained cells by the total number of BrdU-positive cells. **c** Total number of BrdU-positive cells in the SGZ is shown. Data are presented as the mean ± SEM (*n* = 12–15 per group). *p < 0.001 according to Bonferroni’s post hoc test. *GCL* granular cell layer, *SGZ* subgranular zone, *BrdU* bromodeoxyuridine, *DCX* doublecortin, *KS* kososan

We used double immunostaining of BrdU and DCX to quantify the number of DCX-positive cells among the BrdU-labeled cells in the SGZ of the DG. BrdU-labeled cells were visualized as cells with brown colors in the nucleus, whereas DCX-positive cells were detected as cells with gray colors in the soma and dendrites (Fig. 8a). The percentage of BrdU and DCX double-positive cells of water-treated defeated mice in the DG was significantly lower than that of water-treated undefeated mice (Fig. 8b; one-way ANOVA, *F*_(2,38)_ = 30.35, *p* < 0.001; post hoc test, *p* < 0.001), which was significantly rescued by kososan extract treatment (one-way ANOVA, *F*_(2,38)_ = 30.35, *p* < 0. 001; post hoc test, *p* < 0.001). However, the stress-induced reduction in the total number of BrdU-positive cells in the DG (Fig. 8c; one-way ANOVA, *F*_(2,38)_ = 19.75, *p* < 0.001; post hoc test, *p* < 0.001) was not rescued in kososan extract-treated defeated mice. Meanwhile, Ki67, an amplifying cell marker expressed in the SGZ (Additional file 5: Figure S4A; Additional file 6: supplementary method), was decreased in the water-treated defeated mice (Additional file 5: Figure S4B; one-way ANOVA, *F*_(2,53)_ = 3.957, *p* < 0.05; post hoc test, *p* < 0.05), which appeared to be improved by kososan extract treatment, although the difference was not significant.

## Discussion

**Fig. 9:**
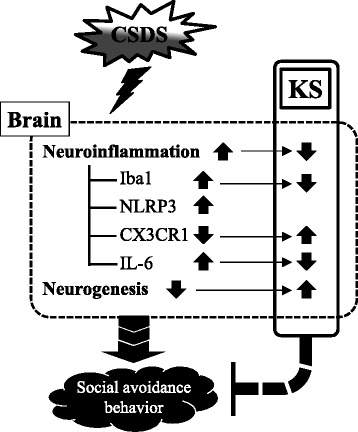
A putative mechanism underlying blockage of social avoidance behavior of kososan extract in socially defeated mice. This study hypothesizes that KS extract prevents a social avoidance behavior as depressive- and anxious-like behaviors by partially mediating neuroinflammation and neurogenesis in the brain of socially defeated mice. *CSDS* chronic social defeat stress, *KS* kososan

A growing body of evidence suggests that stress triggers neuroinflammation, which is linked to the pathogenesis of depression and anxiety. The present study was designed to evaluate the effects of kososan on psychosocial stress-induced behavioral deficits and neuroinflammation. We used the CSDS paradigm as a well-established mouse model of psychosocial stress-induced depression and anxiety and found, for the first time, that kososan extract attenuates a social avoidance behavior, depressive- and anxious-like behaviors, seen in socially defeated mice. This effect was partly mediated by mitigating the enhanced neuroinflammation and disruption of adult neurogenesis caused by CSDS (Fig. 9).

In the present study, avoidance behavior against aggressor mice was observed in the defeated mice. This was indicated by a significant reduction in the SI ratio relative to undefeated control mice and was significantly blocked by kososan extract treatment. Furthermore, these behavioral changes were unlikely to result from alterations in locomotor activity among the groups. Rodents innately have a character of overt sociability, frequently exhibited as sniffing and grooming one another. However, prolonged external aversive stimuli (e.g., defeat stress) can lead to reduced sociability and social avoidance, which is well considered to be a characteristic symptom of depression and anxiety [4, 43]. Therefore, recovery from defeat stress-triggered social avoidance behaviors by kososan extract treatment may represent antidepressant- and/or anxiolytic-like effects under conditions of psychosocial stress.

In defeated mice, greater body weight gain was found relative to undefeated control mice. This is consistent with numerous studies that found that CSDS induces an increase in body weight [52–56], which may result from stress-related hyperphagia or a metabolic aberration. However, the stress-induced increase in body weight was not affected by kososan extract treatment, suggesting that the observed behavioral recovery may be independent of mechanisms that influence body weight.

Accumulating evidence has shown that CSDS enhances microglia-mediated neuroinflammation in rodents [11, 12, 17, 57]. Consistent with these findings, defeated mice from the present study had an increase in Iba1-positive cells and their aggregates [58, 59] and an enhanced inflammatory response after a LPS challenge in isolated microglia. A microglial aggregation is considered to be a sign of microglial activation, thereby contributing to neuroinflammation and subsequent neuronal damages in the brain [59, 60]. It is therefore conceivable that kososan extract-induced reduction in the aggregates corroborates the blocking effect of microglial activation by kososan extract. An increase in CSDS-induced neuroinflammation was also observed as a LPS-stimulated elevation of IL-6 release from ex vivo microglia. These results may reflect stress-induced microglial priming (i.e., a reactive state in preparation for subsequent stimuli like stress or an immune challenge) that may enhance subsequent inflammatory responses [61, 62]. Surprisingly, these CSDS-induced alterations were mitigated by kososan extract treatment, raising a possibility that anti-social avoidant behavior by kososan extract is involved in the suppression of neuroinflammation. Future studies whether behavioral deficits and the corresponding neuroinflammation are blocked in mice administered kososan from after 10-day CSDS exposure will support the possibility. Given our results, the suppressive effect on the enhanced inflammatory response to LPS following treatment with kososan extract may be attributable to the inhibition of microglial priming. In addition, a more recent study has shown that psychological stress-triggered neuroinflammation is strongly linked to crosstalk of microglia with astrocytes, in which astrocyte-derived adenosine triphosphate by stress facilitates microglial activation via the purinergic type 2X7 receptor [63]. Thus, further studies on the role of kososan extract in astrocyte function would be valuable for better understanding the effect of kososan extract on neuroinflammation.

In the present study, wounds were observed in some defeated mice. However, our data on behavioral outcomes and neuroinflammatory responses following CSDS were independent of the extent of wounds (data not shown), which was consistent with previously published studies showing that injuries in the CSDS paradigm did not affect subsequent behaviors and inflammatory responses [64, 65]. Therefore, in the present study, the extent of wounds after CSDS exposure is, at least, unlikely to be a confounding factor in the assessment of social behaviors and neuroinflammation.

M1 and M2 microglial imbalance is deeply linked to neuroinflammation in the CNS [23, 66, 67]. In our study, to further unravel the suppression of neuroinflammation by kososan extract, we examined the regulating effect of kososan extract on the hippocampal M1/M2 microglial balance. In the MOL, CSDS attenuated the anti-inflammatory profile of microglia, as indicated by decreased CX3CR1 (a marker of M2 microglia)-positive cells, an effect that was rescued by treatment with kososan extract. This result suggests that kososan extract mitigates neuroinflammation by increasing the population of anti-inflammatory microglia in the MOL. Conversely, in the SGZ of defeated mice, microglia were more likely to have a pro-inflammatory profile, as indicated by increased NLRP3 (a marker of M1 microglia)-positive and decreased CX3CR1-positive cell numbers, neither of which was affected by kososan extract treatment. These results suggest that the administration of kososan extract failed to attenuate neuroinflammation in the SGZ. Although further studies are necessary to verify the effects of kososan extract on other indicators of microglial phenotypes, our results imply that the effect of kososan extract on neuroinflammation may be due to an increase in the anti-inflammatory M2 phenotype of microglia rather than a decrease in the pro-inflammatory M1 phenotype. Likewise, distinct regional hippocampal differences in the distribution of NLRP3- and CX3CR1-positive cells may reflect a region-specific role of each microglial phenotype in the hippocampal circuitry, neurogenic niche, and behavioral outcomes.

Disruption of adult hippocampal neurogenesis, a well-known process involving the generation and functional integration of newborn cells into brain circuitry [68], plays an important role in the mechanisms by which stress facilitates depression [69, 70]. The hippocampus is a brain region particularly vulnerable to stress and inflammation, and therefore, we examined the impact of CSDS and kososan extract treatment on adult hippocampal neurogenesis. A defeat stress-induced reduction in the survival of newborn cells and their proliferation in the SGZ, as demonstrated by decreased total BrdU- and Ki67-positive cells, was not blunted by kososan extract treatment. However, kososan extract restored the CSDS-induced reduction in neuronal differentiation. These results indicate that kososan extract partially alleviates a stress-triggered disruption of adult hippocampal neurogenesis. A previous report has demonstrated that restraint stress-induced neuroinflammation (i.e., increased activated microglia and NLRP3 expression) was associated with impaired neurogenesis, which is required for depression-like behaviors in mice [71]. CX3CR1 and its ligand fractalkine have also been reported to regulate adult hippocampal neurogenesis in rodents [72]. For example, the disruption of fractalkine/CX3CR1 signaling in adult CX3CR1-deficient mice causes a disturbance in adult hippocampal neurogenesis. Taken together, these findings support our results that a CSDS-induced reduction in CX3CR1-positive cells along with an increase in NLRP3-positive cells in the SGZ may be reciprocally involved in the disruption of neurogenesis, indicated by a reduced number of BrdU/DCX-positive cells. Furthermore, our study found that kososan extract treatment prevented the CSDS-induced disruption of neurogenesis without reversing the reduction in CX3CR1-positive cells and the increase in NLRP3-positive cells caused by stress in the SGZ. In light of our results with previous findings, there appears to be a partial discrepancy in the relationship between cell differentiation, CX3CR1 expression, and NLRP3 expression in the SGZ of kososan extract-treated mice, in a region-specific manner. It is possible that kososan extract may exert direct and/or indirect actions in the recovery of neurogenesis independent of CX3CR1 and NLRP3 profiles in the SGZ, but further study is required to address these hypotheses.

In this study, there were four major limitations and future directions. First, CSDS triggers not only social avoidance behaviors as indicated by this study but also general depressive and anxious states as assessed by forced swimming test (FST) and elevated plus maze test, which are screening tests for depression and anxiety, respectively [43, 73]. In this study, we focused on the behavioral effect of kososan extract on social avoidance as specific depressive- and anxious-like behaviors against aversion induced by CSDS. In another experiment with a slightly different schedule from that of the present study, oral administration of kososan extract for 12 days attenuated a CSDS-triggered increase in immobile behavior, a depressive-like state, in the FST (data not shown). Therefore, future studies on whether kososan extract improves general depressive and anxious states in socially defeated mice are necessary to conclude antidepressant- and anxiolytic-like activities of kososan extract in the CSDS model. Intriguingly, it has also been reported that CSDS enhances hippocampal-dependent fear memory in the contextual fear conditioning paradigm [74] and that the fear memory is closely linked to microglia-mediated neuroinflammation [75–77]. Moreover, CSDS-induced social avoidant behavior can be a possible learned fear against conspecific [78]. Given these findings with our results, it is plausible to assume that kososan-induced reduction in hippocampal neuroinflammation may be a possible contributor to alleviation of the hippocampus-dependent fear memory. Studies about kososan’s effect on fear memory would provide additional evidence for a better interpretation of our findings in this study. Second, it has been reported that CSDS causes neuroinflammation throughout the brain, including the prefrontal cortex and amygdala [79]. This finding is likely to support our data in the ex vivo microglial response to LPS reflecting the inflammatory response profiles of microglia in the whole brain [11, 46, 47, 80]. However, further studies on whether kososan extract affects stress-induced neuroinflammation in other brain regions are needed. Third, kososan’s effects in this study would be preventive rather than therapeutic, because drugs were administered concurrent with exposure to CSDS. Further studies investigating the therapeutic effects of kososan extract in comparison with existing antidepressant treatments [17] using the CSDS paradigm may be useful in the development of therapeutic strategies. Fourth, the active ingredient(s) of kososan extract particularly associated with the anti-inflammatory benefits and behavioral recovery still remains unclear, although there is some evidence indicating that antidepressant-like effects of apigenin [32] and hesperidin [38] are involved in their anti-inflammatory activities. It has been reported that nobiletin, a polymethoxyflavone in the peels of citrus fruits such as *C. unshiu* Markovich (a component herb of kososan), rapidly crosses the blood-brain barrier [81, 82] and that it exerts anti-inflammatory effects in response to LPS-stimulated BV-2 and RAW 264.7 cells (murine microglia and macrophage cell lines, respectively) [83–85]. Nobiletin has also shown antidepressant-like effects in rodent models [40, 41]. Moreover, our preliminary data from in vitro experiments confirmed some anti-inflammatory effect of nobiletin against LPS-challenged microglia isolated from normal and CSDS-exposed adult mice (unpublished data). The extent to which nobiletin contributed to the benefits of kososan in this study requires further investigation.

## Conclusions

This study is the first to report that kososan extract prevents a social avoidant behavior in socially defeated mice. This effect is partially mediated by a reduction in hippocampal neuroinflammation and neurogenesis, presumably by an increased anti-inflammatory, but not decreased pro-inflammatory, phenotype of microglia. Future studies clarifying the mechanisms underlying the anti-neuroinflammatory activity of kososan extract would contribute to the better understanding of the pathology of depression and anxiety and novel therapeutic approaches.

## Additional files

Additional file 1: Figure S1. Time spent in the SI zone and tracking data in the SAT. (A) Mean time spent in the SI zone in the absence and presence of an aggressor is shown. Data are presented as the mean ± SEM (*n* = 18–19 per group). *p < 0.01 and **p < 0.001 according to paired *t* test. (B) Representative tracking data for each group in the absence and presence of an aggressor are presented. SAT, social avoidance test; ND, non-defeated; D, defeated; KS, kososan (PPTX 146 kb)

Additional file 2: Figure S2. Kososan extract per se had no impact on behaviors and neuroinflammation in non-defeated mice. Kososan extract (1.0 g/kg) or distilled water was administered to non-defeated mice orally once daily for 12 consecutive days. SAT (A, B) and Iba1 staining (C) was performed on days 11 and 13, respectively (*n* = 8 per group). In a separate experiment, after isolating microglia from mice treated with kososan extract or distilled water on days 13–15, microglia were stimulated with LPS (0.1 μg/ml) for 18 h, and IL-6 levels in the cultured supernatants were examined (*n* = 6 per group). All data are presented as the mean ± SEM. KS, kososan; DG, dentate gyrus; MOL, molecular layer; GCL, granular cell layer; SGZ, subgranular zone. (PPTX 103 kb)

Additional file 3: Table S1. One-way ANOVA analysis for simple main effects of changes in body weight (PPTX 64 kb)

Additional file 4: Figure S3. The number of Iba1-positive cells (A) or Iba1-positive aggregates (B) found in each of the four subregions of the dentate gyrus. Data are presented as the mean ± SEM (*n* = 18–19 per group). *p < 0.05, **p < 0.01, and ***p < 0.001 according to Bonferroni’s post hoc test. MOL, molecular layer; GCL, granular cell layer; SGZ, subgranular zone; KS, kososan. (PPTX 101 kb)

Additional file 5: Figure S4. Effects of CSDS and kososan extract treatment on the number of Ki67-positive cells in the SGZ. (A) A representative photomicrograph of Ki67-positive (brown-stained) cells in the SGZ. Scale bar = 100 μm. (B) The number of Ki67-positive cells in the SGZ are presented. Data are presented as the mean ± SEM (*n* = 18–19 per group). *p < 0.05 according to Bonferroni’s post hoc test. CSDS, chronic social defeat stress; SGZ, subgranular zone; KS, kososan. (PPTX 13148 kb)

Additional file 6: Supplementary method. (DOC 21 kb)

## Abbreviations

ANOVA: Analysis of variance
BrdU: Bromodeoxyuridine
BSA: Bovine serum albumin
CSDS: Chronic social defeat stress
CX3CR1: CX3C chemokine receptor 1
DCX: Doublecortin
DG: Dentate gyrus
DMEM: Dulbecco’s modified Eagle’s medium
GCL: Granular cell layer
Iba1: Ionized calcium binding adaptor molecule 1
IFN-α: Interferon-alpha
IL-6: Interleukin-6
LPS: Lipopolysaccharide
MOL: Molecular layer
NLRP3: Nod-like receptor family, pyrin domain-containing 3
PBS: Phosphate-buffered saline
PPARγ: Peroxisome proliferator-activated receptor γ
RT: Room temperature
SAT: Social avoidance test
SEM: Standard error of the mean
SGZ: Subgranular zone
SI: Social interaction
