# Supplementary material for: Kososan, a Kampo medicine, prevents a social avoidance behavior and attenuates neuroinflammation in socially defeated mice
Source: J Neuroinflammation. 2017 May 3;14:98. doi: 10.1186/s12974-017-0876-8 (PMC5415730; doi:10.1186/s12974-017-0876-8)
Supplement: Supplementary file 1 — Time spent in the SI zone and tracking data in the SAT. (A) Mean time spent in the SI zone in the absence and presence of an aggressor is shown. Data are presented as the mean ± SEM (n = 18–19 per group). *p < 0.01 and **p < 0.001 according to paired t test. (B) Representative tracking data for each group in the absence and presence of an aggressor are presented. SAT, social avoidance test; ND, non-defeated; D, defeated; KS, kososan (PPTX 146 kb) [file 12974_2017_876_MOESM1_ESM.pptx]

## Slide 1
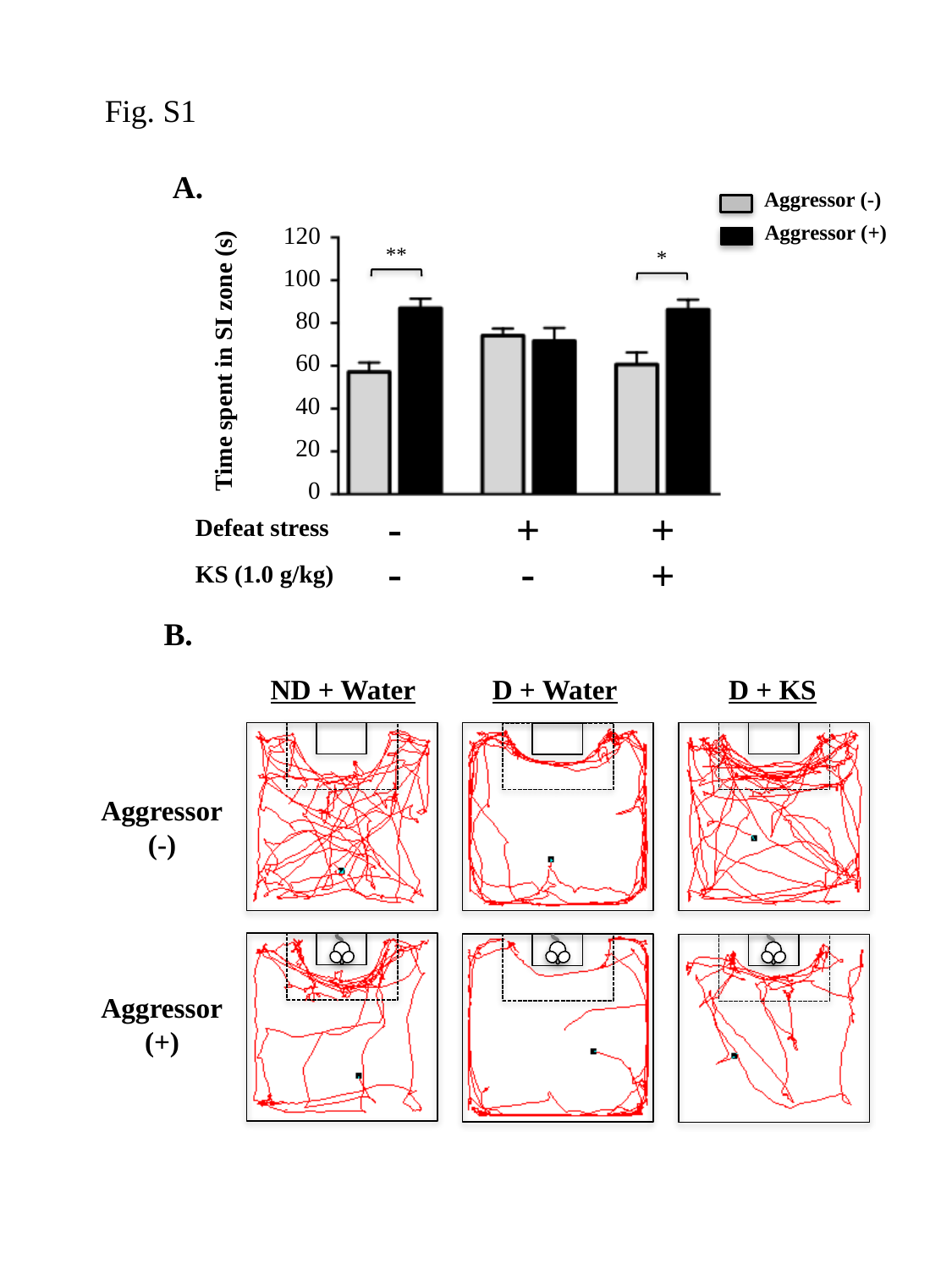

Fig. S1
A.
Aggressor (-)
Aggressor (+)
120
**
*
100
80
Time spent in SI zone (s)
60
40
20
0
-
+
+
Defeat stress
-
-
+
KS (1.0 g/kg)
B.
ND + Water
D + Water
D + KS
Aggressor
(-)
Aggressor
(+)
