# Supplementary material for: Kososan, a Kampo medicine, prevents a social avoidance behavior and attenuates neuroinflammation in socially defeated mice
Source: J Neuroinflammation. 2017 May 3;14:98. doi: 10.1186/s12974-017-0876-8 (PMC5415730; doi:10.1186/s12974-017-0876-8)
Supplement: Supplementary file 2 — Kososan extract per se had no impact on behaviors and neuroinflammation in non-defeated mice. Kososan extract (1.0 g/kg) or distilled water was administered to non-defeated mice orally once daily for 12 consecutive days. SAT (A, B) and Iba1 staining (C) was performed on days 11 and 13, respectively (n = 8 per group). In a separate experiment, after isolating microglia from mice treated with kososan extract or distilled water on days 13–15, microglia were stimulated with LPS (0.1 μg/ml) for 18 h, and IL-6 levels in the cultured supernatants were examined (n = 6 per group). All data are presented as the mean ± SEM. KS, kososan; DG, dentate gyrus; MOL, molecular layer; GCL, granular cell layer; SGZ, subgranular zone. (PPTX 103 kb) [file 12974_2017_876_MOESM2_ESM.pptx]

## Slide 1
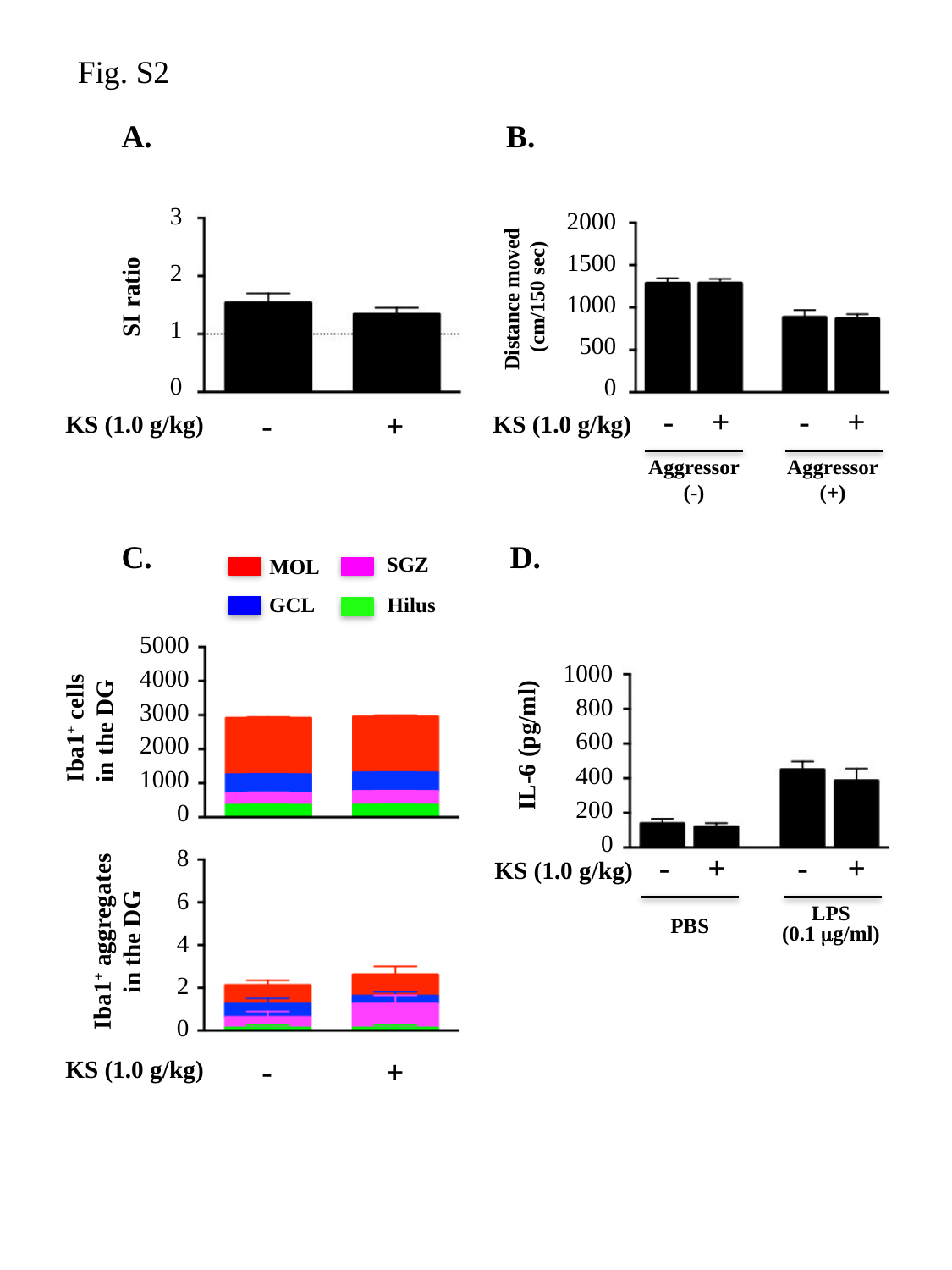

Fig. S2
A.
B.
3
2000
1500
2
Distance moved
 (cm/150 sec)
SI ratio
1000
1
500
0
0
-
+
-
+
-
+
KS (1.0 g/kg)
KS (1.0 g/kg)
Aggressor
(-)
Aggressor
(+)
C.
D.
SGZ
MOL
Hilus
GCL
5000
1000
4000
800
Iba1+ cells
in the DG
3000
600
2000
IL-6 (pg/ml)
400
1000
200
0
0
8
-
+
-
+
KS (1.0 g/kg)
6
LPS
(0.1 mg/ml)
Iba1+ aggregates
in the DG
PBS
4
2
0
-
+
KS (1.0 g/kg)
