# Supplementary material for: Kososan, a Kampo medicine, prevents a social avoidance behavior and attenuates neuroinflammation in socially defeated mice
Source: J Neuroinflammation. 2017 May 3;14:98. doi: 10.1186/s12974-017-0876-8 (PMC5415730; doi:10.1186/s12974-017-0876-8)
Supplement: Supplementary file 3 — One-way ANOVA analysis for simple main effects of changes in body weight (PPTX 64 kb) [file 12974_2017_876_MOESM3_ESM.pptx]

## Slide 1
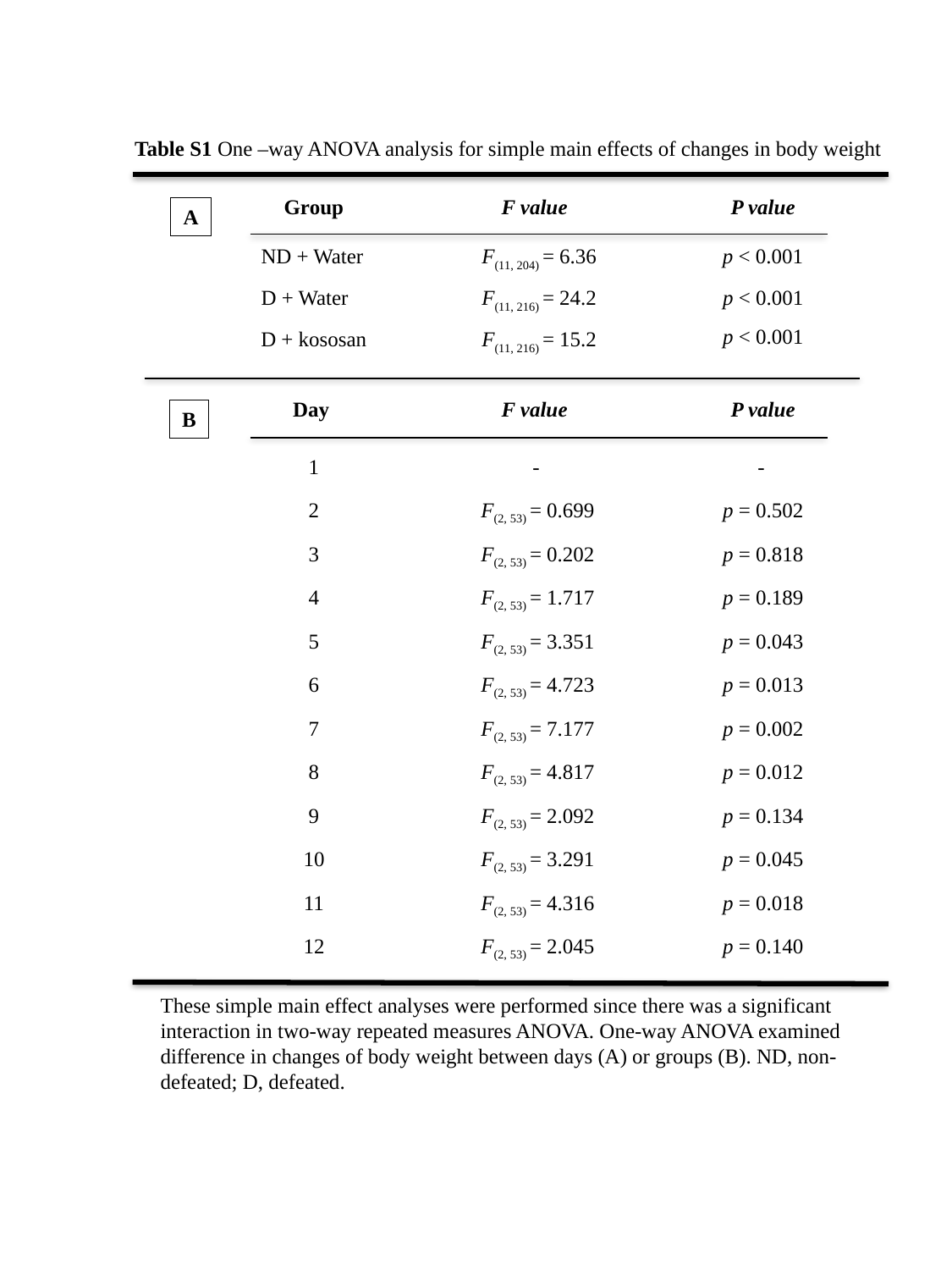

Table S1 One –way ANOVA analysis for simple main effects of changes in body weight
Group
F value
P value
A
ND + Water
F(11, 204) = 6.36
p < 0.001
p < 0.001
D + Water
F(11, 216) = 24.2
p < 0.001
D + kososan
F(11, 216) = 15.2
Day
F value
P value
B
1
-
-
2
F(2, 53) = 0.699
p = 0.502
3
F(2, 53) = 0.202
p = 0.818
4
F(2, 53) = 1.717
p = 0.189
5
F(2, 53) = 3.351
p = 0.043
6
F(2, 53) = 4.723
p = 0.013
7
F(2, 53) = 7.177
p = 0.002
8
F(2, 53) = 4.817
p = 0.012
9
F(2, 53) = 2.092
p = 0.134
10
F(2, 53) = 3.291
p = 0.045
11
F(2, 53) = 4.316
p = 0.018
12
F(2, 53) = 2.045
p = 0.140
These simple main effect analyses were performed since there was a significant interaction in two-way repeated measures ANOVA. One-way ANOVA examined difference in changes of body weight between days (A) or groups (B). ND, non-defeated; D, defeated.
