# Supplementary figures and images for: Kososan, a Kampo medicine, prevents a social avoidance behavior and attenuates neuroinflammation in socially defeated mice
Source: J Neuroinflammation. 2017 May 3;14:98. doi: 10.1186/s12974-017-0876-8 (PMC5415730; doi:10.1186/s12974-017-0876-8)

## Slide 1
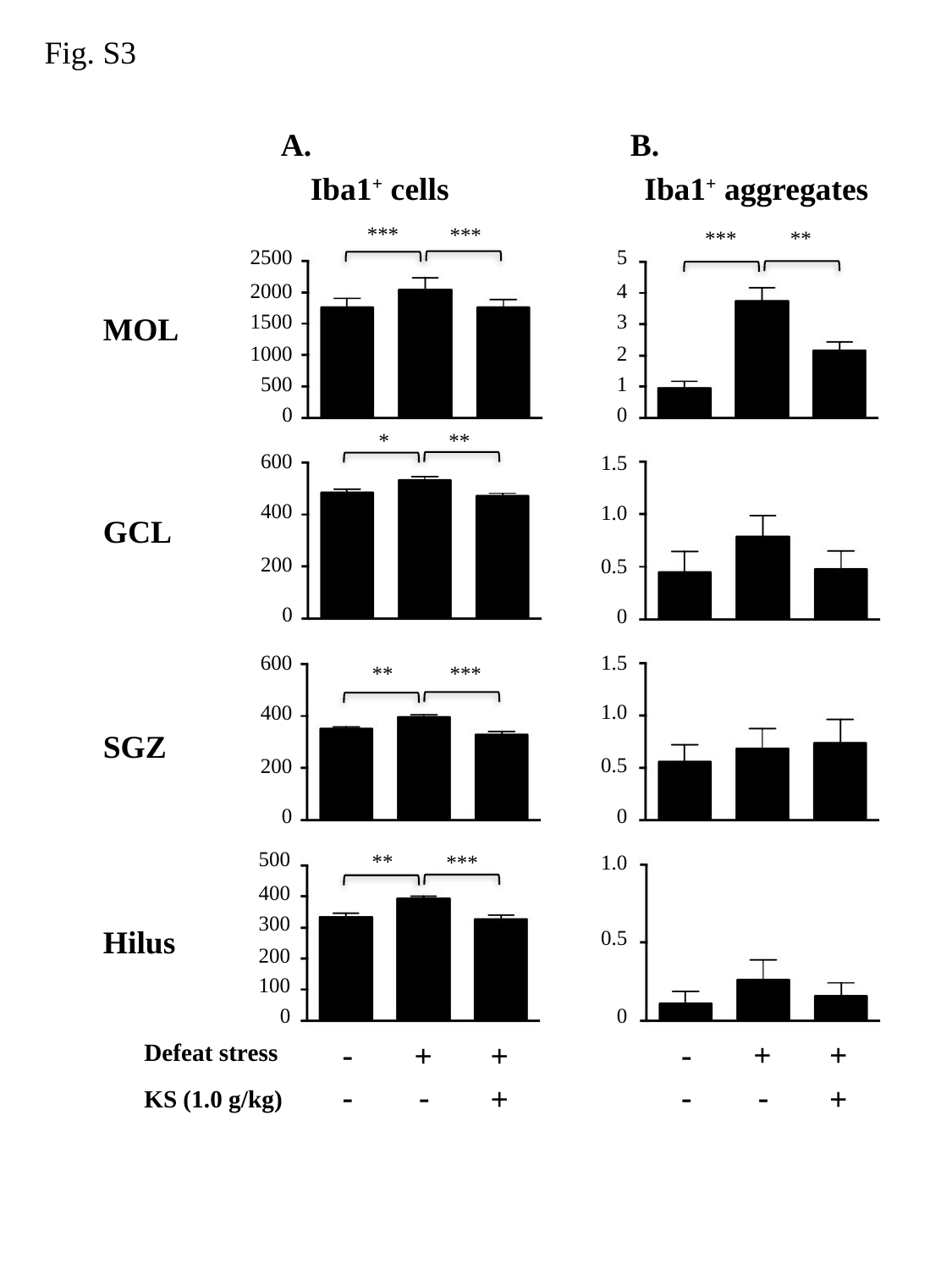

Fig. S3
A.
B.
Iba1+ cells
Iba1+ aggregates
***
***
**
***
2500
5
2000
4
1500
3
MOL
1000
2
500
1
0
0
*
**
600
1.5
400
1.0
GCL
200
0.5
0
0
1.5
600
**
***
1.0
400
SGZ
0.5
200
0
0
500
**
***
1.0
400
300
Hilus
0.5
200
100
0
0
-
-
+
+
+
+
Defeat stress
-
-
-
-
+
+
KS (1.0 g/kg)

Supplement: Supplementary file 4 — The number of Iba1-positive cells (A) or Iba1-positive aggregates (B) found in each of the four subregions of the dentate gyrus. Data are presented as the mean ± SEM (n = 18–19 per group). *p < 0.05, **p < 0.01, and ***p < 0.001 according to Bonferroni’s post hoc test. MOL, molecular layer; GCL, granular cell layer; SGZ, subgranular zone; KS, kososan. (PPTX 101 kb) [file 12974_2017_876_MOESM4_ESM.pptx]

## Slide 1
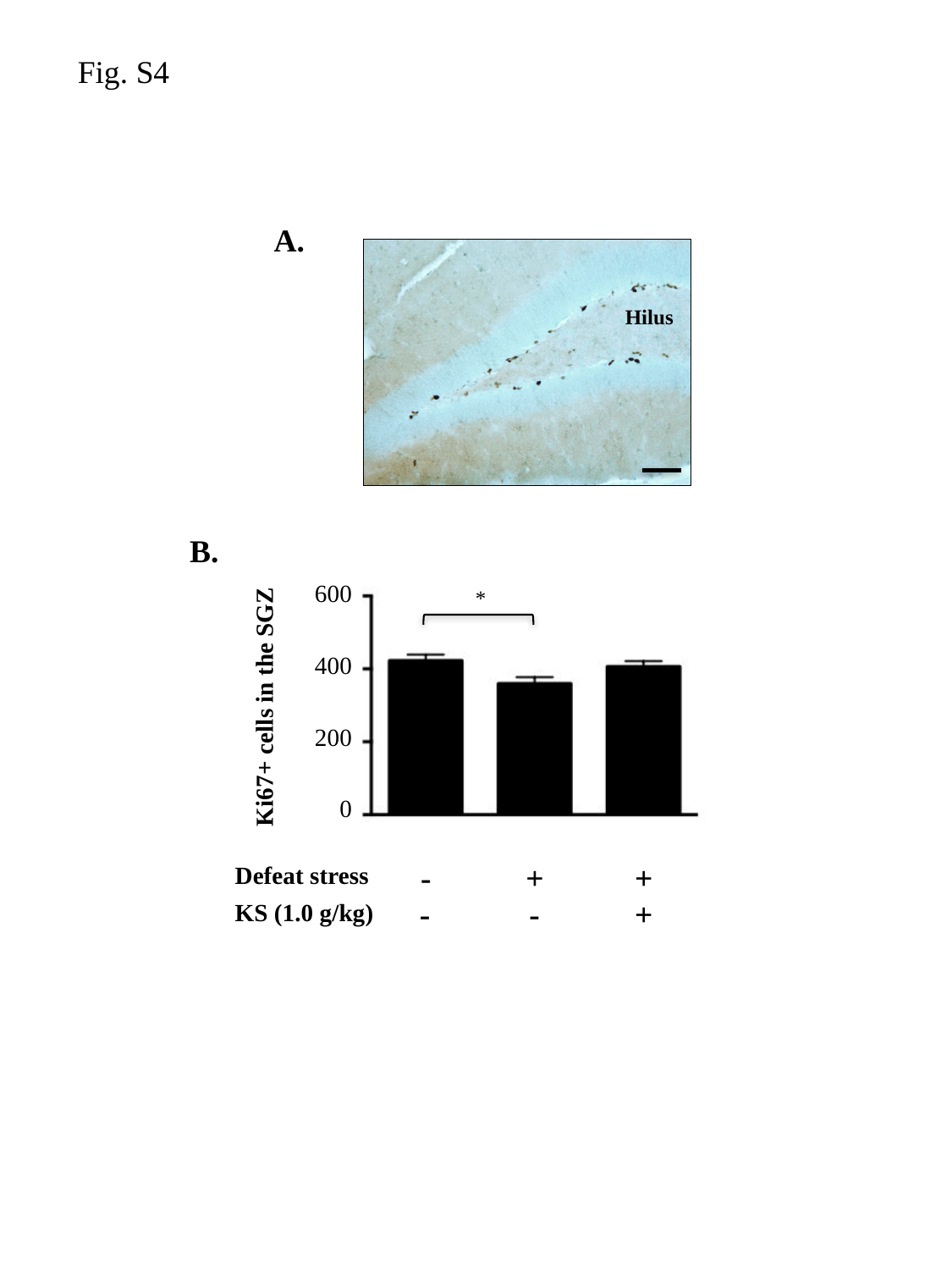

Fig. S4
A.
Hilus
B.
600
*
400
Ki67+ cells in the SGZ
200
0
-
+
+
Defeat stress
-
-
+
KS (1.0 g/kg)

Supplement: Supplementary file 5 — Effects of CSDS and kososan extract treatment on the number of Ki67-positive cells in the SGZ. (A) A representative photomicrograph of Ki67-positive (brown-stained) cells in the SGZ. Scale bar = 100 μm. (B) The number of Ki67-positive cells in the SGZ are presented. Data are presented as the mean ± SEM (n = 18–19 per group). *p < 0.05 according to Bonferroni’s post hoc test. CSDS, chronic social defeat stress; SGZ, subgranular zone; KS, kososan. (PPTX 13148 kb) [file 12974_2017_876_MOESM5_ESM.pptx]
