# Supplementary material for: Kososan, a Kampo medicine, prevents a social avoidance behavior and attenuates neuroinflammation in socially defeated mice
Source: J Neuroinflammation. 2017 May 3;14:98. doi: 10.1186/s12974-017-0876-8 (PMC5415730; doi:10.1186/s12974-017-0876-8)
Supplement: Supplementary file 6 — Supplementary method. (DOC 21 kb) [file 12974_2017_876_MOESM6_ESM.doc]

Supplementary method

**Immunohistochemistry for Ki67**

Ki67 staining was performed using the same method for Iba1 staining described in the Methods, except for the primary antibody used [rabbit anti-Ki67 polyclonal antibody (1:500; Abcam, Cambridge, UK)].
